# Functional Study of *Amorpha fruticosa WRKY20* Gene in Response to Drought Stress

**DOI:** 10.3390/ijms241512231

**Published:** 2023-07-31

**Authors:** Danni Li, Baoxiang Gu, Chunxi Huang, Jiayi Shen, Xin Wang, Jianan Guo, Ruiqiang Yu, Sirui Mou, Qingjie Guan

**Affiliations:** 1 Key Laboratory of the Ministry of Education for Ecological Restoration of Saline Vegetation, College of Life Sciences, Northeast Forestry University, Harbin 150040, China; lidanni@nefu.edu.cn (D.L.);; 2 Northeast Asia Biodiversity Research Center, Northeast Forestry University, Harbin 150040, China

**Keywords:** *Amorpha fruticosa*, WRKY transcription factor, drought stress, tobacco genetic transformation, stress resistance

## Abstract

The WRKY gene family in plants regulates the plant’s response to drought through regulatory networks and hormone signaling. *AfWRKY20* (MT859405) was cloned from *Amorpha fruticosa* (*A. fruticosa*) seedlings using RT-PCR. The binding properties of the AfWRKY20 protein and the W-box (a DNA cis-acting element) were verified both in vivo and in vitro using EMSA and Dual-Luciferase activity assays. RT-qPCR detected that the total expression level of *AfWRKY20* in leaves and roots was 22 times higher in the 30% PEG6000 simulated drought treatment compared to the untreated group. Under the simulated drought stress treatments of sorbitol and abscisic acid (ABA), the transgenic tobacco with the *AfWRKY20* gene showed enhanced drought resistance at the germination stage, with significantly increased germination rate, green leaf rate, fresh weight, and root length compared to the wild-type (WT) tobacco. In addition, the superoxide dismutase (SOD) activity, chlorophyll content, and Fv/Fm ratio of *AfWRKY20* transgenic tobacco were significantly higher than those of the WT tobacco under natural drought stress, while the malondialdehyde (MDA) content and 3,3′-diaminobenzidine (DAB) and nitroblue tetrazolium (NBT) staining levels were lower. The expression levels of oxidation kinase genes (*NbSOD*, *NbPOD*, and *NbCAT*) in transgenic tobacco under drought stress were significantly higher than those in WT tobacco. This enhancement in gene expression improved the ability of transgenic tobacco to detoxify reactive oxygen species (ROS). The survival rate of transgenic tobacco after natural drought rehydration was four times higher than that of WT tobacco. In summary, this study revealed the regulatory mechanism of *AfWRKY20* in response to drought stress-induced ABA signaling, particularly in relation to ROS. This finding provides a theoretical basis for understanding the pathways of WRKY20 involved in drought stress, and offers genetic resources for molecular plant breeding aimed at enhancing drought resistance.

## 1. Introduction

Drought is a prominent abiotic stress factor that poses a threat to the growth and development of plants, resulting in a significant decrease in crop yield [1]. Such stressful conditions lead to the accumulation of osmotic and oxidative regulators, which in turn induce the expression of stress-related genes [2]. The efficiency of photosynthesis determines a plant’s ability to withstand natural drought stress. Chlorophyll fluorescence parameters are key to the study of photosynthesis [3]. Under drought stress conditions, the process of light absorption and electron transport in photosynthesis can result in the accumulation of ROS not only in chloroplasts [4], but also in plants. This accumulation can damage the cell membrane system and cause oxidative stress. Fortunately, plants can not only utilize various mechanisms to reduce ROS production but also acquire ROS scavenging systems to protect themselves [5]. Plants transfer stress signals through signal transduction pathways in vivo and regulate the enzymatic antioxidant system, which includes SOD, peroxidase (POD), catalase (CAT), and other antioxidant enzymes. This system helps remove excessive ROS components in cells [6]. In addition, for ROS accumulated in chloroplasts, non-photochemical quenching, photorespiration, and the Calvin–Benson–Bassham cycle (CBB) can dissipate the energy of excess electrons in chloroplasts and decrease the production of ROS [7,8]. Moreover, drought stress triggers the accumulation of the plant hormone ABA through hyperosmotic signaling. This accumulation leads to the development of multiple ABA signaling pathways in plants. These pathways can promote adaptive responses to drought stress. Even in the absence of water stress, ABA inhibits plant stem and root growth [9].

Thus, plants have developed various molecular mechanisms, including signal transduction and gene expression, to adapt to abiotic stresses. Evolved transcription factors (TFs), such as the CBF [10], ERF [11], BHLH [12], bZip [13], ZFP [14], MYB [15], NAC [16], C2H2 [17], Dof [18], HSF [19], and WRKY [20] families, can regulate gene network expression to combat environmental stresses. The WRKY gene family is a group of plant-specific transcription factors (TFs) that play important roles in various aspects, including plant defense response [21], plant growth and development [22], and the regulation of leaf senescence [23]. WRKY TFs are characterized by a conserved domain, which includes a WRKYGQK motif at the N-terminus and a zinc finger motif at the C-terminus [24]. According to the number of conserved WRKY domains and the structural characteristics of zinc finger motifs, they can be divided into three groups: Group Ⅰ, Group Ⅱ and Group Ⅲ. Group Ⅱ can be further divided into five subgroups: Ⅱa, Ⅱb, Ⅱc, Ⅱd and Ⅱe [25]. When plants are exposed to external stimuli, WRKY TFs are regulated by a cascade of defense signaling networks. They can recognize and bind to the W-box sequence [TTGAC(C/T)] present in the promoter region of the target gene, thereby participating in the regulatory network and enhancing the defense ability of plants [26].

WRKY TFs also play key roles in the transcriptional regulation and signal transduction processes in plants. They extensively regulate the expression changes of target genes in various physiological programs and are involved in various stress pathways [27]. Certain *Arabidopsis* WRKY TFs function as positive regulators in the pathway of ABA-mediated stomatal closure [28]. In addition, the *WRKY20* gene in soybean is sensitive to ABA in terms of regulating stomatal closure. This sensitivity can enhance the plant’s tolerance to drought stress [29]. In addition, WRKY TFs can also positively regulate drought resistance by improving ABA biosynthesis; for example, the WRKY TF ZmWRKY79 in maize has been demonstrated to have this capability [30]. In the signal transduction pathways mediated by jasmonate (JA) and salicylic acid (SA), the WRKY70 TF is activated by SA, while its expression is suppressed by JA [31]. In terms of regulating plant growth and development, VvWRKY2 is specifically expressed in lignified cells of young grape stems. This expression affects the lignin biosynthesis pathway, which in turn impacts xylem development [32].

There is still a need to further explore the potential of WRKY TFs with different domains in various species. Recent transcriptome studies have shown that herbaceous plants, such as wheat (*Triticum aestivum* L.) [33], licorice (*Glycyrrhiza glabra* L.) [34], broad bean (*Vicia faba* L.) [35], and millet (*Panicum miliaceum*) [36] upregulate WRKY TFs in response to drought stress. WRKY TFs in various woody plants, including Myrothamnus flabellifolia [37], and oil palm (*Elaeis guineensis* Jacq.) [38], also respond to drought stress. However, functional studies of WRKY TFS in the woody plant *A. fruticosa* are limited. *A. fruticosa* is a perennial leguminous woody plant with strong adaptability. It can survive in adverse conditions, including cold, windy, and saline-alkali environments in northeastern China. It can also be used as a plant for greening, soil improvement, windbreak, and forest stabilization [39,40]. In addition, the roots, stems, leaves, and fruits of *A. fruticosa* not only possess medicinal properties for reducing dampness and swelling, but they also have significant economic and practical value [41,42]. More importantly, *A. fruticosa* has a high tolerance to drought stress [43].

Although studies on the drought resistance of *A. fruticosa* WRKY TFs are limited, transcriptome sequencing analysis of *A. fruticosa* has shown that the *AfWRKY20* gene is upregulated in response to drought-induced expression [44]. In this study, the *AfWRKY20* gene was cloned from the transcriptome sequencing of *A. fruticosa* under drought stress using RT-PCR technology. Subsequently, bioinformatics analysis, phylogenetic tree construction, and subcellular localization verification were conducted. The binding properties of *AfWRKY20* and W-box were verified both in vivo and in vitro through EMSA and Dual-Luciferase activity assays. The expression pattern of *AfWRKY20* in response to abiotic stress was investigated through qRT-PCR analysis. To investigate the resistance of *AfWRKY20* transgenic tobacco lines to drought stress at different growth stages, the study first subjected them to sorbitol stress and ABA stress as a means of simulating drought stress treatment. The study then measured phenotypic data, including germination rate, green leaf rate, fresh weight, and root length of the *AfWRKY20* transgenic tobacco lines during the germination stage. Secondly, in this experiment, one-month-old and two-month-old *AfWRKY20* transgenic tobacco lines were subjected to natural drought stress. The chlorophyll fluorescence parameters and the survival rate of the one-month-old *AfWRKY20* transgenic tobacco lines after rewatering were measured. To investigate the potential of *AfWRKY20* in enhancing the detoxification of ROS in tobacco, the study measured the activity of SOD, MDA content, levels of DAB and NBT staining, as well as the expression levels of oxidation kinase genes (*NbSOD*, *NbPOD*, and *NbCAT*) in *AfWRKY20* transgenic tobacco lines after 2 months of natural drought. This study provides a reference for the role of *AfWRKY20* in regulating reactive oxygen species in the ABA signaling response induced by drought stress. It also provides an experimental and theoretical basis for understanding the drought tolerance function of this TF by elucidating the molecular mechanism of *AfWRKY20* in enhancing drought stress regulation.

## 2. Results

### 2.1. Cloning and Bioinformatics Analysis of AfWRKY20

**Figure 1:**
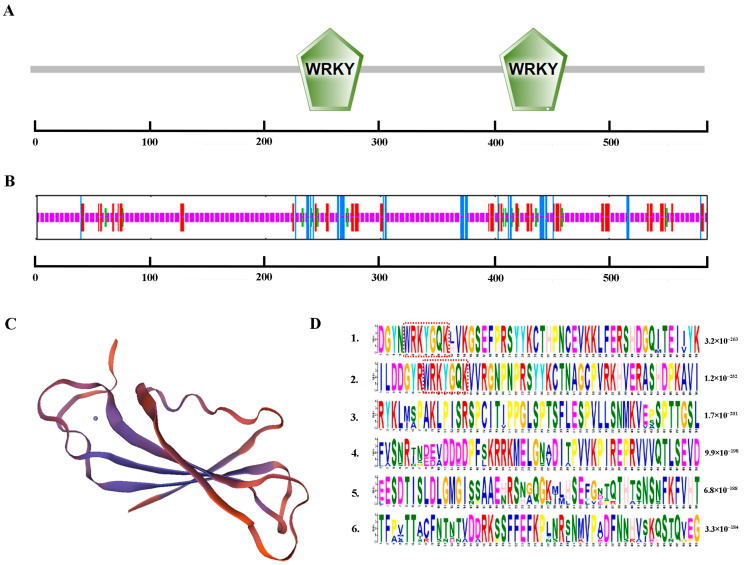
Results of bioinformatics analysis of AfWRKY20. (**A**) Primary structure of the AfWRKY20 protein. (**B**) The secondary structure of the AfWRKY20 protein is depicted, with the α-helix shown in blue, the β-turn in green, the extended strand in red, and the irregular coil in purple. (**C**) Tertiary structure of the AfWRKY20 protein. (**D**) The AfWRKY20 motif consists of a conserved domain, with the two red-dashed rectangular regions representing the two WRKY domains.

Total RNA was extracted from *A. fruticosa* leaves (Figure S1A), and a 20 μL cDNA reverse transcription product was prepared. The target band, which was approximately 1758 bp, was then amplified (Figure S1B). The recombinant plasmid pMD18-T-*AfWRKY20* was successfully constructed and sent for sequencing. The NCBI BLAST alignment showed that the nucleotide sequence was correct. AfWRKY20 (c194398, graph_c0) was predicted by the SMART online website to contain two WRKY domains (231–289 aa, 407–466 aa), a C2C2-type zinc finger protein, and a C2H2-type zinc finger protein (Figure 1A). The predictions for the secondary and tertiary structures were consistent (Figure 1B,C). In addition, Plant-mPLoc predicted that AfWRKY20 is localized in the nucleus (Figure S2B). Using the MEME online website to predict the two WRKY domains of *A. fruticosa* WRKY20, it was found that the two WRKY domains were highly conserved (Figure 1D).

### 2.2. Sequence Alignment and Phylogenetic Analysis

**Figure 2:**
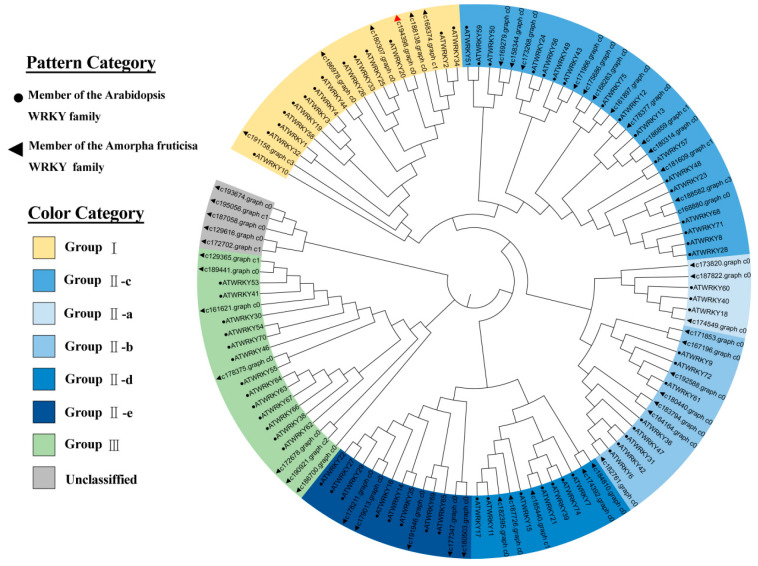
Construction of phylogenetic trees for drought stress WRKY transcription factors in *A. fruticosa* and *Arabidopsis* WRKY transcription factors. The red triangle represents WRKY20 of *A. fruticosa*. Triangles indicate WRKY transcription factors in the *A. fruticosa* drought transcriptome. Circles indicate WRKY transcription factors in *Arabidopsis*. The classification of WRKY transcription factors is distinguished by different colors.

Phylogenetic relationships between *Arabidopsis* WRKYs and WRKYs in the transcriptome sequencing of *A. fruticosa* under drought stress were analyzed. Under drought stress, 51 WRKY TFs of *A. fruticosa* were identified from the transcriptome sequencing data. These TFs were categorized into four main groups: Group Ⅰ, Group Ⅱ, Group Ⅲ, and an unclassified group. These groups contained 6, 33, 7, and 5 *A. fruticosa* WRKY TFs, respectively. Group Ⅱ was further subdivided into five subgroups (Ⅱc, Ⅱa, Ⅱb, Ⅱd, and Ⅱe) containing 13, 3, 7, 5, and 5 *A. fruticosa* WRKY TFs, respectively. *AfWRKY20* (c194398, graph_c0) contained two WRKY domains, and its zinc finger domain is of the C2H2 type. Therefore, it was classified as part of the first group. Seventy *Arabidopsis* WRKY transcription factors were divided into three groups: Group I contained 14 *Arabidopsis* WRKY transcription factors, Group Ⅱ contained 43 *Arabidopsis* WRKY transcription factors, and Group Ⅲ contained 13 *Arabidopsis* WRKY transcription factors. Group Ⅱ was further subdivided into five subgroups (Ⅱc, Ⅱa, Ⅱb, Ⅱd and Ⅱe). They contained 17, 3, 8, 7, and 8 *Arabidopsis* WRKY TFs, respectively. The branches of the phylogenetic tree showed that *Arabidopsis* WRKY20 and *A. fruticosa* WRKY20 in Group I were closely related (Figure 2).

**Figure 3:**
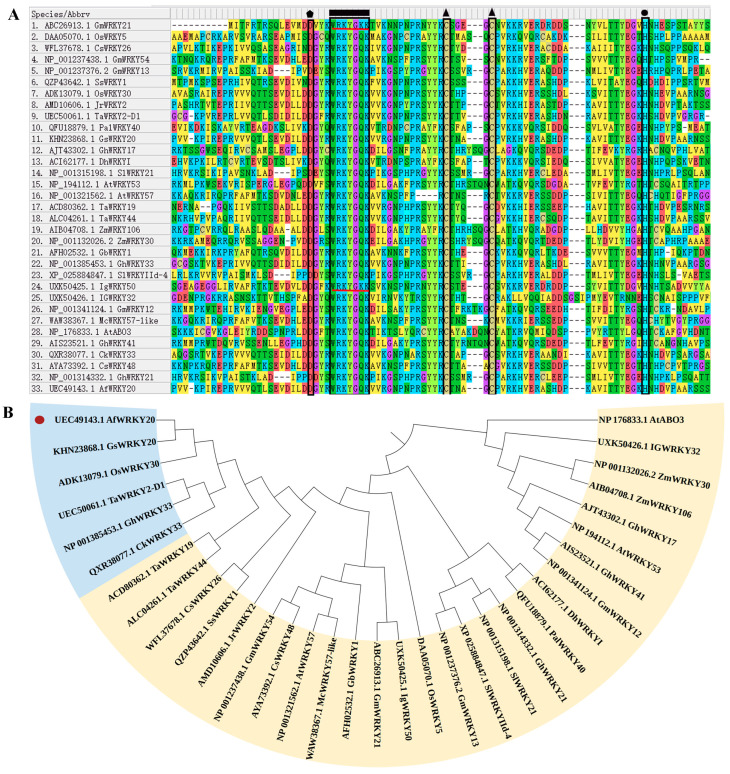
(**A**) The amino acid sequences of the drought-resistant WRKY transcription factors of *A. fruticosa* WRKY20 and various species were compared using MEGA 7.0 software. The WRKY domain, C, and H residues in the zinc finger motif are represented by rectangles, triangles, and circles, respectively. The region of the red line represents WRKYGKK, which is not the regular WRKY domain motif (WRKYGQK).The pentagon represents the site as aspartic acid. (**B**) Phylogenetic trees were constructed for *A. fruticosa* WRKY20 and drought-resistant WRKYs from various species. The red circles indicate AfWRKY20. The blue regions represent drought-resistant WRKY transcription factors with a very high homology to AfWRKY20. The yellow region represents the drought-resistant WRKY transcription factors that show homology to AfWRKY20.

In addition, a sequence comparison of *A. fruticosa* WRKY20 with 32 reported drought-resistant WRKY transcription factors from different species showed that, except for GmWRKY, IgWRKY50 had a unique WRKYGKK residue, while the rest had WRKYGQG, C, and H residues (Figure 3A). Phylogenetic tree construction revealed that AfWRKY20 was most closely related to GSWRKY20 (Figure 3B). In addition, to explore the species origin of *A. fruticosa* WRKY20, a phylogenetic tree was constructed to compare it with different species. The results showed that *A. fruticosa* WRKY20 was closely related to the WRKY20 of peanut and red bean (Figure S2A).

### 2.3. Subcellular Localization of AfWRKY20 Protein

**Figure 4:**
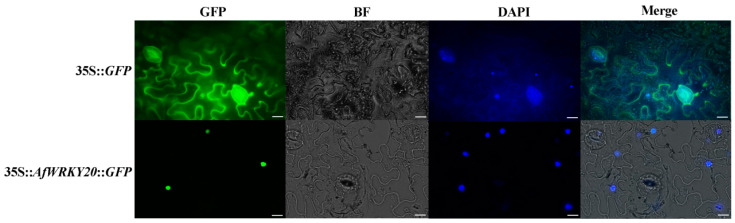
Subcellular localization of AfWRKY20 in tobacco cells. 35S-*GFP* was used as the control group. 35S-*AfWRKY20*-*GFP* was used as the experimental group. Ruler: 20 μm.

The control group (35S-*GFP*) was observed to be localized in the nucleus, cytoplasm, and cell membrane using confocal fluorescence microscopy (Olympus, Tokyo, Japan). In order to investigate the subcellular localization of AfWRKY20, the fusion protein of green fluorescent protein (GFP)—AfWRKY20 was expressed transiently in *N. benthamiana* leaves, driven by the 35S promoter. AfWRKY20 localizes in the nucleus and overlaps with the signal of the nuclear-specific dye DAPI (Figure 4). The results of the AfWRKY20 protein experiment were consistent with the subcellular localization of the AfWRKY20 protein predicted by Plant-mPLoc, further confirming that the AfWRKY20 protein is located in the nucleus.

### 2.4. Analysis of Binding Properties of AfWRKY20 Protein and W-Box Cis-Acting Elements

**Figure 5:**
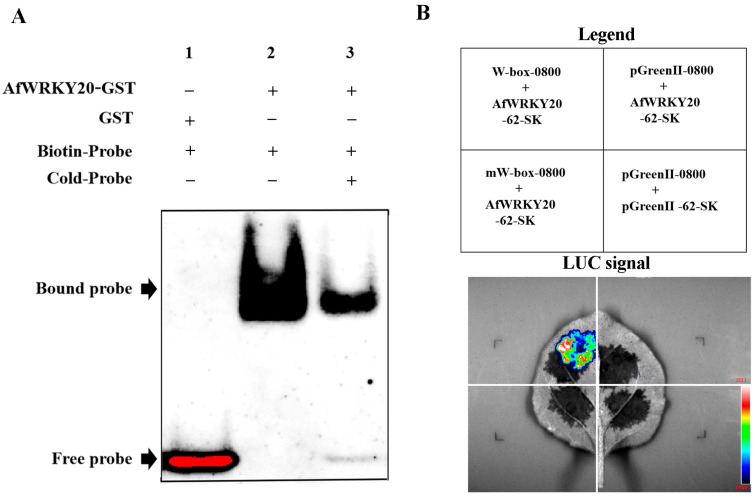
(**A**) Binding of the GST-AfWRKY20 fusion protein to the W-box element. (1) GST+ protein and a biotin-labeled oligonucleotide probe (Biotin-Pr) were added. (2) Adding GST-AfWRKY20 fusion protein and biotin-Pr. (3) Adding GST-AfWRKY20 fusion protein, biotin-Pr and unlabeled oligonucleotide probe (Cold-Pr). (**B**) Dual-LUC analysis of W-box and mW-box elements.

In order to investigate the characteristics of AfWRKY20 coding protein binding to the DNA cis-element W-box of the WRKY TF both in vivo and in vitro, we utilized the electrophoretic mobility shift assay (EMSA) and Dual-LUC assay to validate the binding characteristics. EMSA kits were used to detect the gel-blocking signals of biotin-labeled probes and GST-AfWRKY20 fusion proteins (Figure 5A). The results showed that no band transfer was observed when biotin-labeled probes and GST proteins were added to the mixture. This finding suggested that the GST protein did not bind to the DNA probe. However, when the fusion protein (GST-AfWRKY20) was mixed with biotin-Pr, a DNA-binding band was detected. The addition of competing probes weakened the composite signal of AfWRKY20 binding to the W-box and enhanced the signal of the free probe. This result indicates that the fusion protein is unable to bind to the cis-element W-box after binding to the TTGAC sequence of the DNA probe. Collectively, these experimental results confirmed that the protein encoded by *AfWRKY20* possesses the functional characteristics of a WRKY TF that binds to the W-box in vitro.

To confirm whether the AfWRKY20 protein can bind to W-box elements in vivo, this study was performed using a Dual-LUC reporter assay in *N. benthamiana* tobacco. When mW-box-0800 was co-transformed with the *AfWRKY20*-62-SK vector, no LUC luminescence signal was observed. When W-box-0800 was co-transformed with the *AfWRKY20*-62-SK vector, a clear LUC luminescence signal was generated (Figure 5B). Thus, the AfWRKY20 protein is a representative WRKY protein that exhibits a specific binding affinity for the W-box element, as observed through in vitro and in vivo experiments.

### 2.5. Characterization of AfWRKY20 Gene Expression

**Figure 6:**
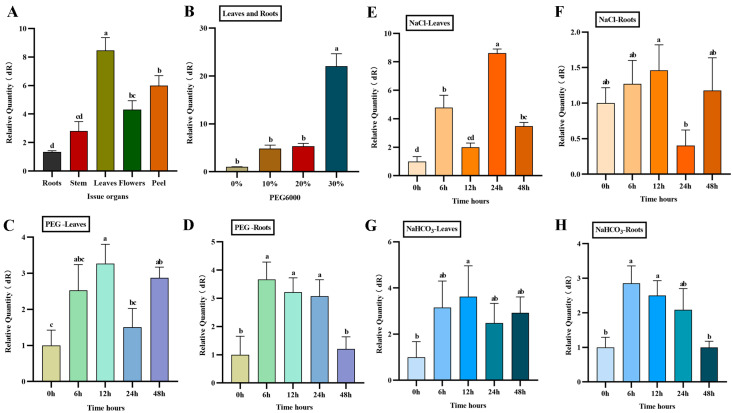
Expression characteristics of the *AfWRKY20* gene in tissues and organs under PEG6000-induced stress. (**A**) Tissues and organs: roots, stems, leaves, flowers, and peel. (**B**) Expression of *AfWRKY20* in leaves and roots under 0%, 10%, 20%, and 30% PEG6000 stress. (**C**) Expression of the *AfWRKY20* gene in leaves under 30% PEG6000 stress at 0, 6, 12, 24, and 48 h. (**D**) Expression of the *AfWRKY20* gene in roots under 30% PEG6000 stress at 0, 6, 12, 24, and 48 h. (**E**) Expression of the *AfWRKY20* gene in leaves exposed to 150 mmol/L NaCl stress at 0, 6, 12, 24, and 48 h. (**F**) Expression of the *AfWRKY20* gene in roots under 150 mmol/L NaCl stress at 0, 6, 12, 24, and 48 h. (**G**) Expression of the *AfWRKY20* gene in leaves exposed to 30 mmol/L NaHCO_3_ stress at 0, 6, 12, 24, and 48 h. (**H**) Expression of the *AfWRKY20* gene in roots under 30 mmol/L NaHCO_3_ stress at 0, 6, 12, 24, and 48 h. Error bars represent standard errors of three biological replicates, with significant differences at the *p* < 0.05 level. Lowercase letters (a, b, c, etc.) represent statistical differences with *p* < 0.05.

RT-qPCR was used to detect the expression of *AfWRKY20* in various tissues and organs of *A. fruticosa*. The results showed that the gene was expressed in roots, stems, leaves, and flowers. Its expression level was highest in leaves and lowest in roots (Figure 6A). Under different concentrations (0%, 10%, 20%, and 30%) of PEG6000 to simulate drought stress, the expression of *AfWRKY20* increased with the increase in PEG6000 concentration. In addition, the overall expression level of *AfWRKY20* in leaves and roots significantly increased under treatment with 30% PEG6000 compared to the control group, reaching 22 times that of the control group (Figure 6B).

The effects of three stress conditions (30% PEG6000, 150 mmol/L NaCl, and 30 mmol/L NaHCO_3_) on the expression of *AfWRKY20* in the roots and leaves of *A. fruticosa* were analyzed. The results of the study showed that the expression of the *AfWRKY20* gene increased most significantly at 12 h of *A. fruticosa* leaf treatment compared to the control under 30% PEG6000 treatment (Figure 6C). However, the expression of the *AfWRKY20* gene was significantly decreased after 48 h of root treatment (Figure 6D). Under 150 mmol/L NaCl stress, the expression level of *AfWRKY20* in the leaves and roots of *A. fruticosa* exhibited unstable fluctuations. The expression of the *AfWRKY20* gene was 9-fold higher in leaves after 24 h of stress (Figure 6E). The expression of *AfWRKY20* was significantly reduced in roots after being stressed for 24 h (Figure 6F). Under the treatment of 30 mmol/L NaHCO_3_, the expression level of *AfWRKY20* initially increased in the leaves and roots of *A. fruticosa*, but then decreased. At 12 h, the expression of *AfWRKY20* in leaves reached its highest level, which was 3.7-fold higher than that of the control (Figure 6G). At 6 h after *A. fruticosa* root treatment, the expression of *AfWRKY20* peaked, which was 2.7 times higher than that of the control (Figure 6H).

### 2.6. Genetic Transformation and Drought Resistance Analysis of AfWRKY20 Transgenic Tobacco

#### 2.6.1. Acquisition of *AfWRKY20* Transgenic Tobacco

The PCR molecular identification of T0 generation *AfWRKY20* transgenic tobacco lines showed that the target band size of *AfWRKY20* transgenic tobacco lines (#4, #5, #6, #7) was identical to that of the positive control plasmid pBI121-*AfWRKY20*-GFP (CK+). However, the negative control did not exhibit any band. This indicated that *AfWRKY20* had been successfully integrated into the genomic DNA of tobacco (Figure S3A). The expression of the transgenic tobacco lines was analyzed using RT-qPCR. The results showed that the expression level of the *AfWRKY20* gene was significantly higher in transgenic tobacco lines 5, 6, and 7 compared to the WT (Figure S3B).

The seeds of WT and T3 transgenic tobacco lines were sown on 1/2 MS medium and a kanamycin (50 mg/L) resistant medium. The results showed that the seeds of both the WT and *AfWRKY20* transgenic lines grew normally on 1/2 MS medium. It was proven that the seeds from both the experimental group and the control group were able to germinate normally (Figure S3C(a)). Screening on Kana (50 mg/L) resistant medium showed that all WT unrooted and *AfWRKY20* transgenic tobacco lines (#5, #6, and #7) grew normally, indicating that pure and *AfWRKY20* transgenic tobacco lines had been obtained (Figure S3C(b)). Therefore, T3 generation *AfWRKY20* transgenic tobacco #5, #6, and #7, which are both pure and highly expressed, were selected as the experimental materials for subsequent drought resistance stress.

#### 2.6.2. Tolerance Analysis of *AfWRKY20* Transgenic Tobacco at Germination Stage under Sorbitol and ABA Simulated Drought Stress

**Figure 7:**
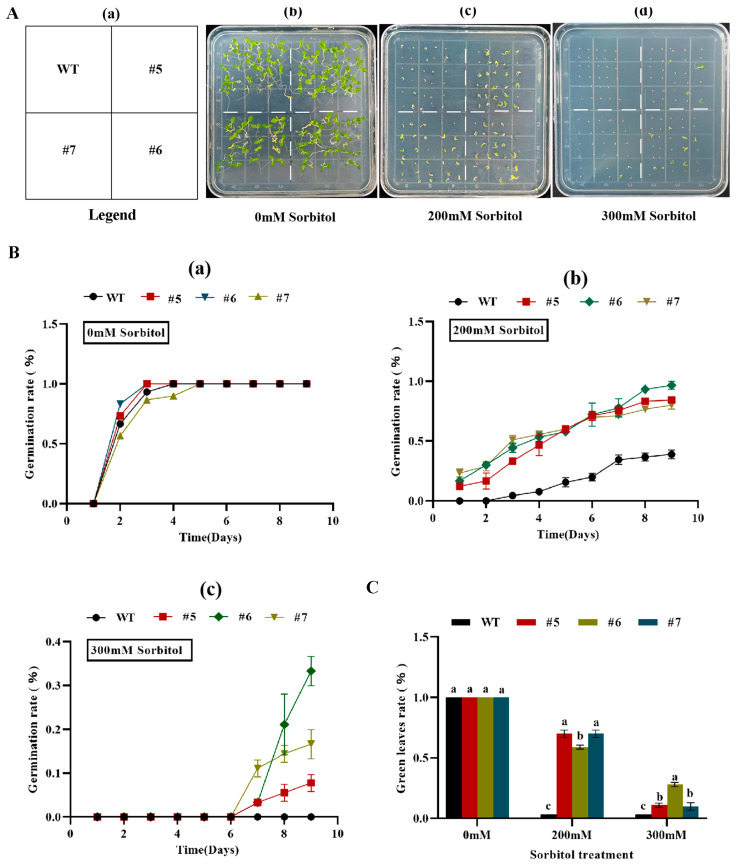
Analysis of the green leaf rate and germination rate of *AfWRKY20* overexpression lines under sorbitol stress at various concentrations. (**A**) Sorbitol stress phenotypes at different concentrations. (**a**) Schematic diagram of tobacco placement. (**b**–**d**) Germination phenotypes of tobacco under 0 mM, 200 mM, and 300 mM sorbitol stress.(**B**) Measurement of germination rate. (**a**–**c**) Germination trend of tobacco under 0 mM, 200 mM, and 300 mM sorbitol stress. (**C**) Measurement of the percentage of green leaves. Error bars indicate the standard errors of three biological replicates, which are significantly different at the *p* < 0.05 level. Lowercase letters (a, b, c, etc.) represent statistical differences with *p* < 0.05.

When transgenic tobacco seeds containing *AfWRKY20* were treated with different concentrations of sorbitol (0, 200, and 300 mM) for 15 days, the germination rate of the transgenic tobacco lines was higher compared to that of the WT tobacco lines. In addition, as the stress-inducing concentration of sorbitol was gradually increased, the germination advantage of the transgenic lines became more apparent (Figure 7B). When exposed to sorbitol stress for 20 days, the transgenic tobacco lines exhibited a significantly higher rate of green leaf retention compared to the WT. The most significant difference was observed under 200 mM sorbitol (Figure 7C). At the same time, transgenic tobacco seeds with *AfWRKY20* at different stress-inducing concentrations of ABA (0, 2, 2.5, 3, and 5 μM) during the germination stage showed a higher germination rate and green leaf rate compared to WT tobacco (Figure S5). These results suggest that the *AfWRKY20* gene may enhance the drought stress tolerance of tobacco seeds during the germination stage under stress conditions simulated by sorbitol and ABA.

**Figure 8:**
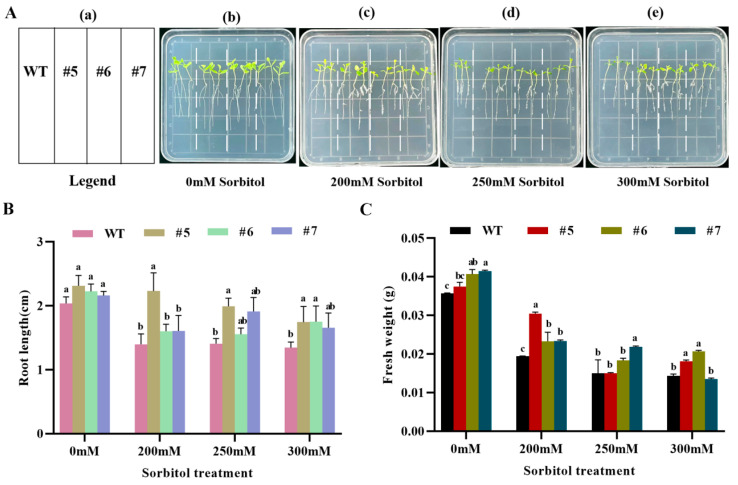
Analysis of fresh weight and root length of *AfWRKY20* overexpression tobacco lines under sorbitol stress at various concentrations. (**A**) The phenotypes of tobacco under sorbitol stress. (**a**) Schematic diagram of tobacco placement. (**b**–**e**) Phenotypic map of tobacco under 0 mM, 200 mM, 250 mM, and 300 mM sorbitol stress. (**B**) Measurement of plant root length. (**C**) Measurement of fresh weight of plants. Lowercase letters (a, b, c, etc.) represent statistical differences with *p* < 0.05.

Statistical analysis of root length (Figure 8B) and fresh weight (Figure 8C) of WT and *AfWRKY20* transgenic tobacco at the trifoliate stage after 10 days of stress induced with different concentrations of sorbitol (0, 200, 250, and 300 mM) showed that the fresh weight and root length of transgenic tobacco lines were significantly greater than those of WT tobacco lines. The most significant difference was observed at 200 mM sorbitol. After 15 days of stress with different concentrations of ABA (0, 5, 7.5, and 10 μM) at the trifoliate stage, both WT and *AfWRKY20* transgenic tobacco lines showed significant improvements in root length (Figure S6B) and fresh weight (Figure S6C) compared to the WT line. These results suggest that the *AfWRKY20* gene may regulate the growth and development of tobacco under conditions of drought stress simulated with sorbitol and ABA. This regulation may enhance tobacco’s tolerance to drought stress.

#### 2.6.3. Analysis of Photosynthetic Characteristics of *AfWRKY20* Transgenic Tobacco Pot Seedlings under Natural Drought Stress

**Figure 9:**
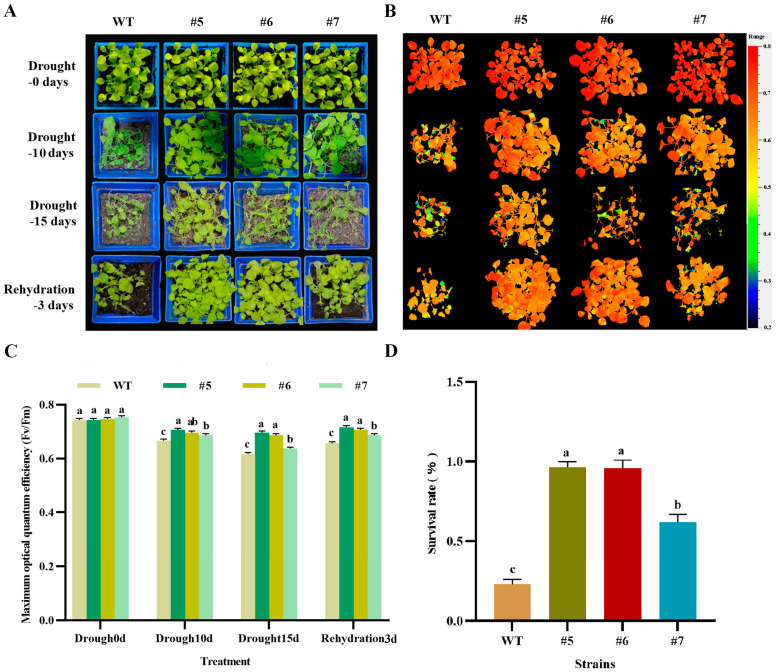
Analysis of photosynthetic characteristics of *AfWRKY20* transgenic tobacco pot seedlings under natural drought stress. (**A**) Phenotyping of tobacco plants after 0, 10, and 15 days of natural drought treatment, followed by 3 days of rehydration. (**B**) Images of chlorophyll fluorescence Fv/Fm in transgenic tobacco and the WT tobacco under drought stress treatment, with a cursor range of 0.2–0.8. (**C**) Fv/Fm values of WT and transgenic tobacco were measured at 0, 10, and 15 days of drought treatment, as well as 3 days after rehydration. (**D**) Survival rate of tobacco plants after 3 days of rehydration. Lowercase letters (a, b, c, etc.) represent statistical differences with *p* < 0.05.

One-month-old T3 generation *AfWRKY20* transgenic tobacco pot seedlings were subjected to natural drought conditions for 0, 10, and 15 days before being rewatered for 3 days. Images were taken under natural light (Figure 9A), and then chlorophyll fluorescence was imaged using the FluorCam open chlorophyll fluorescence imaging system in Fv/Fm mode (Figure 9B). The photosynthetic characteristics of transgenic tobacco pot seedlings and wild-type pot seedlings were analyzed under natural drought stress. The results showed that the trend of F0 of WT was more pronounced than that of the transgenic lines during the 15-day drought treatment (Figure S4A). The Fm value of the WT decreased compared to transgenic lines 5 and 6, but was higher compared to transgenic line 7 (Figure S4B). The decrease in QL was more pronounced in the WT than in the transgenic strain (Figure S4C). NPQ increased more slowly in the early stages of drought stress (0–10 days) and showed a larger decrease in the later stages (10–15 days) compared to the transgenic lines (Figure S4D). These results indicated that the PSII reaction center was more damaged in the WT tobacco than in the *AfWRKY20* transgenic tobacco.

The transgenic tobacco pot seedlings were subjected to drought treatment for 0, 10, and 15 days, followed by rewatering for 3 days. The wild-type tobacco plants were also included in the experiment. The plants were photographed under natural light (Figure 9A), revealing that the growth and development of the transgenic tobacco plants were superior to those of WT tobacco. A chlorophyll fluorescence imaging system was also used to capture chlorophyll fluorescence images at Fv/Fm (range: 0.2–0.8) (Figure 9B). It was observed that the fluorescence color of the drought-treated WT tobacco was not as red as that displayed by the transgenic tobacco. The chlorophyll fluorescence imaging system was used to quantitatively analyze the maximum optical quantum efficiency (Fv/Fm) after 0, 10, and 15 days of drought treatment, followed by 3 days of rewatering. The results showed that the Fv/Fm values of both the WT and transgenic tobacco decreased significantly after drought treatment compared to untreated tobacco. However, the Fv/Fm value of the transgenic tobacco was still significantly higher than that of the WT tobacco (Figure 9C). Although the Fv/Fm values of both the WT and transgenic tobacco recovered after 3 days of rehydration, the Fv/Fm value of the transgenic tobacco remained higher than that of the WT tobacco (Figure 9C). In addition, the survival rate of transgenic tobacco after rehydration was significantly higher than that of the WT tobacco (Figure 9D). These results suggest that *AfWRKY20* can reduce the photosynthetic damage caused by drought stress and participate in the molecular mechanism of tobacco photosystem II, thereby enhancing the drought resistance of tobacco.

#### 2.6.4. Determination of Physiological Indices of Drought in *AfWRKY20* Transgenic Tobacco

**Figure 10:**
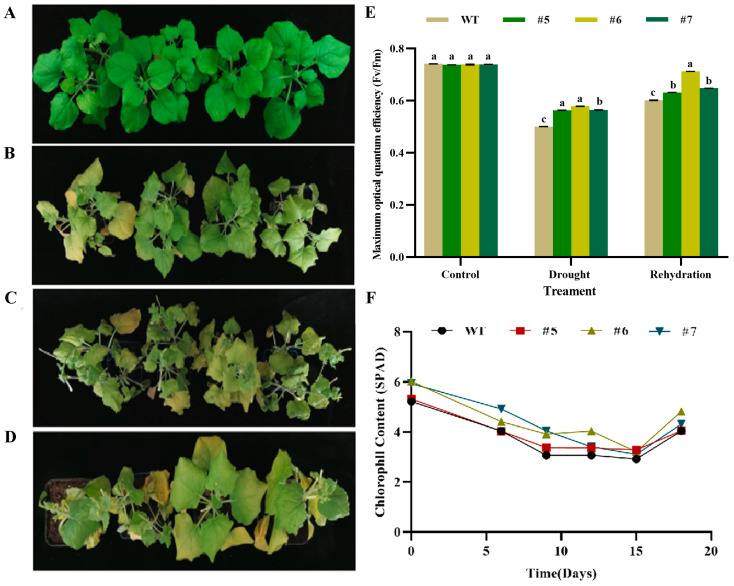
Phenotypic changes and photosynthetic characteristics of *AfWRKY20* transgenic tobacco under natural drought stress. (**A**) Phenotype of tobacco on day 0 of drought treatment. (**B**) Phenotypes of tobacco plants after 10 days of drought treatment. (**C**) Phenotypes of tobacco plants after 15 days of drought treatment. (**D**) The phenotypes of tobacco after 3 days of rewatering. (**E**) The Fv/Fm values comparing *AfWRKY20* overexpression tobacco with the WT tobacco on day 0 of drought treatment, after 15 days of drought treatment, and 3 days after rewatering. Lowercase letters (a, b, c, etc.) represent statistical differences with *p* < 0.05. (**F**) Changes in chlorophyll content in tobacco during drought treatment and subsequent rewatering.

The T3 generation of *AfWRKY20* transgenic lines grown for 2 months were subjected to 0, 10, and 15 days of natural drought treatment before being rewatered for 3 days. Before treatment, all plants exhibited a consistent growth state (Figure 10A). However, during the drought treatment, the transgenic tobacco exhibited distinct phenotypic changes compared to the WT tobacco. At 10 days of drought treatment, both the transgenic and the WT tobacco leaves exhibited slight wilting and yellowing. However, the changes were more noticeable in the WT tobacco (Figure 10B). After 15 days of drought treatment, the growth condition of transgenic tobacco leaves was significantly superior to that of WT leaves (Figure 10C). At 3 days of rewatering after the drought treatment, the tobacco plants that were overexpressed exhibited improved resilience (Figure 10D) and significantly higher Fv/Fm values (Figure 10E) compared to the WT tobacco. The chlorophyll content (SPAD) of transgenic tobacco was higher than that of WT tobacco throughout the natural drought treatment (Figure 10F).

**Figure 11:**
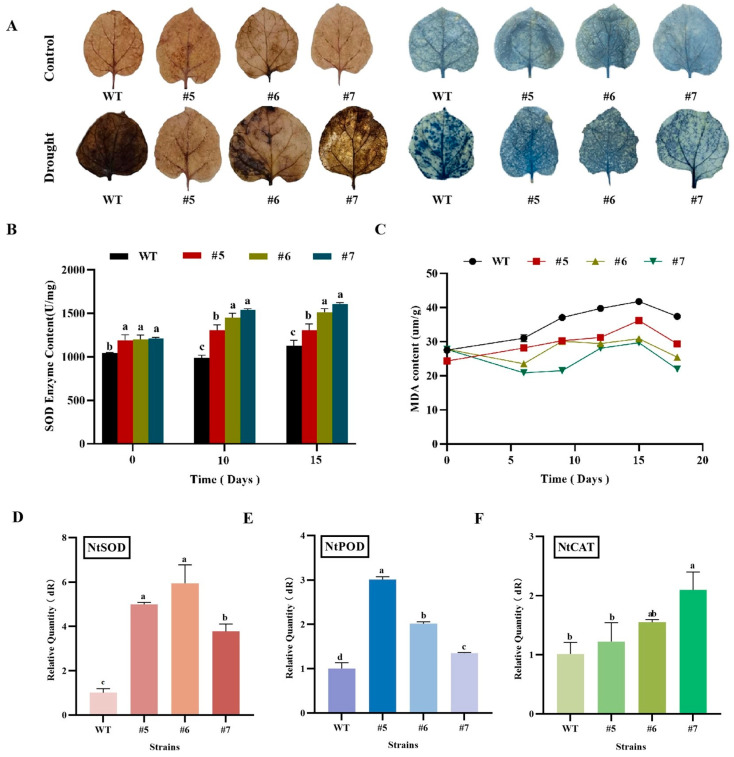
Analysis of oxygen free radicals in tobacco plants under natural drought conditions. (**A**) Histochemical analysis of DAB and NBT in tobacco. (**B**) Determination of the SOD content in tobacco under natural drought conditions. (**C**) Determination of MDA content in tobacco under natural drought conditions. (**D**) Real-time quantitative expression of *NbSOD* during drought stress. (**E**) Real-time quantitative expression of *NbPOD* during drought stress. (**F**) Real-time quantitative expression of *NbCAT* during drought stress. Lowercase letters (a, b, c, etc.) represent statistical differences with *p* < 0.05.

Histochemical analysis using DAB and NBT as ROS indicators revealed deeper staining in the WT tobacco leaves than in transgenic leaves under drought stress. This finding indicates that the WT tobacco accumulated more hydrogen peroxide and superoxide anions during drought stress than transgenic tobacco, resulting in severe leaf damage (Figure 11A). SOD activity reflects the plant’s ability to scavenge oxygen free radicals. After the drought treatment, the SOD content in the plants gradually increased with the extension of drought stress, reaching its peak at 10 days of drought (Figure 11B). In addition, the MDA content in the transgenic plants was significantly lower than that in the WT tobacco (Figure 11C). The gene expression levels of *NbSOD* (Figure 11D), *NbPOD* (Figure 11E), and *NbCAT* (Figure 11F) in transgenic tobacco were significantly higher than those in the WT tobacco, as determined by real-time fluorescence quantitative PCR. In transgenic tobacco, this increase in gene expression resulted in an enhancement of antioxidant enzyme levels in the plant, thereby improving the plant’s ability to scavenge ROS. These experimental results indicate that the overexpression of the *AfWRKY20* gene enhances tobacco’s tolerance to drought stress. This study provides a molecular basis for further investigating the potential of *AfWRKY20* in enhancing stress tolerance in plants.

## 3. Discussion

Drought has a significant impact on plant growth and development [45]. WRKY TFs are critical for regulating plant responses to abiotic stresses [46,47]. Related studies have shown that a new WRKY transcription factor, MuWRKY3 (*Macrotyloma uniflorum Lam.* Verdc.), can enhance drought resistance in transgenic peanut (*Arachis hypogaea* L.) plants [48]. In addition to the search for novel WRKY transcription factors, further investigation is needed to understand the resistance function of WRKY transcription factors in various species. Therefore, *AfWRKY20* (c194398, graph_c0) was screened during transcriptome sequencing of *A. fruticosa* under drought stress to investigate the molecular mechanisms regulating drought stress. Bioinformatics analysis revealed that *AfWRKY20* contains two WRKY domains (231–289 aa, 407–466 aa) with a high degree of conservatism and a C2H2-type zinc finger protein. According to the classical classification criteria, this protein was classified into Group I, indicating its close relationship with the growth, development, and stress tolerance of nuclear organisms (Figure 1) [49].

Based on the construction of the phylogenetic tree, *A. fruticosa* WRKY20 was found to be most closely related to *Arabidopsis* WRKY20 (Figure 2). Related studies have shown that species with high homology have similar gene functions [50]. *AtWRKY20* coregulates the ABA signaling pathway with ABSCISIC ACID-INSENSITIVE 5 (ABI5). Thus, it plays an important role in biological processes such as seed germination, dormancy, anthocyanin synthesis, and response to stress [51]. To further explore the correlation between *A. fruticosa* WRKY20 and drought-resistant WRKY transcription factors in other species, a phylogenetic tree was constructed. The results revealed a significant similarity between *AfWRKY20* and *GsWRKY20* (Figure 3). *GsWRKY20* plays an important role in enhancing drought tolerance and regulating ABA signaling. Therefore, the present study hypothesized that *AfWRKY20* enhances drought stress tolerance in tobacco by regulating the ABA signaling pathway.

In this study, validation experiments were performed to determine the subcellular localization of the pBI121::*AfWRKY20*::*GFP* fusion expression vector. The experimental results were consistent with the software’s prediction that the protein would be localized in the nucleus. Therefore, *AfWRKY20* may play a role in regulating cell signaling molecules (Figure 4) [52]. The binding properties of the AfWRKY20 TF and the DNA cis-element W-box were confirmed through EMSA experiments (Figure 5A) and double LUC experiments (Figure 5B). The W-box mainly exists in the promoter regions of resistance genes associated with disease and insect resistance, drought, low temperature, saline-alkali, and other factors. It can regulate plant resistance by mediating hormone signal transduction pathways. The molecular mechanism of the AfWRKY20 protein regulating drought stress may be similar to that of SbWRKY30. Both of them induce the expression of drought tolerance genes by binding to W-box elements in the promoters of drought tolerance genes in plants, thereby improving the plants’ drought tolerance [53].

When *A. fruticosa* was treated with different concentrations (0%, 10%, 20%, and 30%) of PEG6000 to simulate drought stress, the total expression level of *AfWRKY20* in leaves and roots was 22 times higher in the 30% PEG6000 treatment compared to the untreated group (Figure 6B). In addition, the effects of three different stress conditions (30% PEG6000, 150 mmol/L NaCl, and 30 mmol/L NaHCO_3_) on the expression level of *AfWRKY20* in the roots and leaves of *A. fruticosa* were analyzed. Under 30% PEG6000 treatment, the expression of the *AfWRKY20* gene increased most significantly at 12 h of *A. fruticosa* leaf treatment compared to the control. Under the stress of 150 mmol/L NaCl, the expression levels of *AfWRKY20* in the leaves and roots of *A. fruticosa* exhibited unstable fluctuations (Figure 6E,F). Under the treatment of 30 mmol/L NaHCO_3_, the expression levels of *AfWRKY20* in the leaves and roots of *A. fruticosa* showed an overall trend of initially increasing and then decreasing (Figure 6G,H). *AfWRKY20* was differentially expressed under these three distinct stress conditions. Therefore, it is speculated that the *AfWRKY20* gene may regulate multiple stress pathways. For example, *AtWRKY53* can respond to both drought stress and salt stress, and *AtWRKY6* can regulate both mechanical damage and oxidative stress [54].

When tobacco plants overexpressing *AfWRKY20* were exposed to stress induced by different concentrations of sorbitol (Figure 7 and Figure 8) and ABA (Figures S5 and S6), the germination rate, green leaf rate, root length, fresh weight, and other phenotypes were significantly higher compared to those of WT tobacco. Our results suggest that *AfWRKY20* may regulate the growth and development of tobacco by influencing the ABA signaling pathway, thereby enhancing its drought resistance.

Analysis of the photosynthetic characteristics of transgenic seedlings under drought stress indicated that *AfWRKY20* could reduce the damage caused by stress to photosynthesis and enhance drought stress tolerance. This finding was confirmed by measuring the chlorophyll fluorescence parameters F0, Fm, Fv/Fm, QL, and NPQ of transgenic tobacco plants under natural drought stress (Figure S4). After rehydration, the Fv/Fm values and survival rate of the transgenic plants were significantly higher than those of the WT plants (Figure 9 and Figure 10). This suggests that AfWRKY20 plays a crucial role in regulating photosystem damage caused by drought stress [55].

*AfWRKY20* overexpression in tobacco under natural drought stress resulted in lower MDA content (Figure 11C) and reduced DAB and NBT staining (Figure 11A). These findings indicate a decrease in ROS in the transgenic lines. At the same time, it resulted in higher SOD enzyme activity in vivo (Figure 11B). The expression levels of *NbSOD* (Figure 11D), *NbPOD* (Figure 11E), and *NbCAT* (Figure 11F) in transgenic tobacco were significantly higher than those in the WT tobacco, as determined by real-time fluorescence quantitative PCR. This indicates an enhancement in the content of antioxidant enzymes in the plant. It is speculated that the regulatory mechanism of AfWRKY20 may be similar to that of maize ZmWRKY40. This mechanism involves reducing the levels of ROS in transgenic lines during drought stress. This is achieved by enhancing the activities of POD and CAT, which ultimately enhances the drought resistance of the transgenic lines [56].

The above results suggest that the AfWRKY20 TF can act as a node in the drought stress signaling pathway through ROS balance and ABA signaling pathways, which is similar to the mechanism of XsWRKY20 as a positive regulator. This study revealed the regulatory mechanism of *AfWRKY20* in response to drought stress-induced ABA signaling, involving ROS [57]. These findings lay the foundation for further studies on the mechanisms through which *AfWRKY20* enhances drought tolerance, providing genetic resources for molecular plant breeding aimed at developing drought-resistant varieties.

## 4. Materials and Methods

### 4.1. Plant Material

Seeds of *A. fruticosa* Linn were obtained from Wu Songquan’s research group at Yanbian University, and those of *N. benthamiana* were acquired from Bu Qingyun’s research group at the Northeast Institute of Geography and Agroecology, Chinese Academy of Sciences.

### 4.2. Strains, Vectors, and Reagents

*Escherichia coli* (Top10 competent) and *Agrobacterium tumefaciens* (EHA105) were preserved in the Key Laboratory of Northeast Saline Alkaline Vegetation Restoration and Reconstruction, which is under the Ministry of Education.

PMD18-T vector was acquired from TaKaRa, and the Gateway series entry vector PQBV3, plant expression vector pGWB18, and pBI121-MCS-*GFP* plant expression vector were obtained from the Key Laboratory of Northeast Saline Vegetation Restoration and Reconstruction, Ministry of Education. pGreenⅡ0800-LUC and pGreenⅡ62-SK-LUC carrier were gifted by the Institute of Geography, Chinese Academy of Sciences. T4-DNA ligase, 2×Ex-Taq DNA polymerase, and real-time fluorescent dye 2×SYBR Green qPCR Master Mix were supplied by TaKaRa. Acetosyringone was purchased from Solarbio Biologics, and D-luciferin was obtained from PROMEGA Company. RNA extraction kits, gel recovery kits, and plasmid extraction kits were purchased from Kangwei Company (Beijing, China). Domestic analytical reagents were used for all other experiments.

### 4.3. Cloning and Bioinformatics Analysis of AfWRKY20 Gene

#### 4.3.1. Gene Cloning

In the transcriptome data of *A. fruticosa* under 20% PEG6000 simulated drought stress, after mining of highly differentially expressed genes under drought stress, c194398 and graph_c0 were found to respond to drought stress. Therefore, according to the CDS sequence of c194398, graph_c0, the gene was designated as *AfWRKY20* (accession number: MT859405). Specific primers, *AfWRKY20* F1 and *AfWRKY20* R1 (*AfWRKY20* F1/R1 in Table S1) were designed using Primer 5.0 software. The full-length sequence of *AfWRKY20* was amplified by RT-PCR and then ligated into the pMD18-T vector. The recombinant vector was transformed into TOP10 through heat shock transformation. The plasmids in the successful bacterial solution were then identified by PCR and extracted using the Beijing Kangwei Century Plasmid Extraction Kit. The recombinant plasmids were then sent to Kumei company for sequencing.

#### 4.3.2. Bioinformatics Analysis

The SMART (SMART: Main page (embl-heidelberg.de)) [58] was used to predict the primary structure of AfWRKY20, while the SOPMA (NPSP@: SOPMA secondary structure prediction (ibcp.fr (accessed on 1 October 2022))) was used to predict its secondary structure. The SWISS-MODEL (The SWISS-MODEL Interactive Workspace (expasy.org (accessed on 1 October 2022))) [59] was used to predict the tertiary structure of AfWRKY20. The Plant-mPLoc (Plant-mPLoc server (sjtu.edu.cn (accessed on 1 October 2022))) [60] was used to predict the subcellular localization of AfWRKY20. The MEME (Introduction-MEME Suite (meme-suite.org (accessed on 1 October 2022))) [61] was used to predict the conservation of the WRKY domain in AfWRKY20.

### 4.4. Sequence Alignment and Construction of Phylogenetic Trees

Multiple sequence alignments were performed on WRKY amino acid sequences using ClustalW in MEGA 7.0 with default parameters. A phylogenetic tree was constructed using the neighbor-joining (NJ) method with MEGA 7.0 software. These parameters were used in the NJ method: bootstrap (1000 replicates), complete deletion, and amino: p distance [62]. This setup was employed for constructing all phylogenetic trees in this paper.

In order to compare the phylogenetic relationships of 70 *Arabidopsis* protein WRKYs and 51 drought-stressed *A. fruticosa* WRKYs, all 121 WRKY protein sequences were divided into four groups (Ⅰ, Ⅱ, Ⅲ, and unclassified). Group Ⅱ was further divided into five subgroups (Ⅱa, Ⅱb, Ⅱc, Ⅱd, Ⅱe) [63]. In order to investigate the phylogenetic relationships between *AfWRKY20* and other drought-resistant WRKY transcription factors, we compared the amino acid sequences of 32 reported drought-resistant WRKY transcription factors with *AfWRKY20* and constructed a phylogenetic tree. Additionally, a phylogenetic tree was constructed to explore the homology between *AfWRKY20* and different species of WRKY.

### 4.5. Characterization of AfWRKY20 Gene Expression

To investigate the differential expression of *AfWRKY20* in various tissues and organs of *A. fruticosa*, seeds of *A. fruticosa* were cultured in 96-well plates using the hydroponic method for 4 weeks. Different tissues (root, stem, leaf, and flower) were selected from the growth and development of *A. fruticosa* to extract total RNA. To investigate the gene expression characteristics of *AfWRKY20* under different stress conditions, *A. fruticosa* plants with the same growth rate as *A. fruticosa* were selected. These plants were then treated with 30 mmol/L NaHCO_3_, 150 mmol/L NaCl, and 30% PEG6000. The roots and leaves were collected at 0, 6, 12, 24, and 48 h. Afterwards, *A. fruticosa* seedlings were treated with various concentrations of PEG6000 (0%, 10%, 20%, and 30%) for 48 h, and then leaves and roots were collected. Expression of *AfWRKY20* gene at 0 h under adversity stress was used as a control. The total RNA was extracted from the samples mentioned above, and the concentration of RNA was examined. One microgram (μg) of total RNA was reverse transcribed into complementary DNA (cDNA) and diluted 100-fold as a template for fluorescence quantitative PCR. After conducting RT-qPCR analysis using primers for the internal reference gene (*AfTubu.*F/R in Table S1) and specific primers for *AfWRKY20* (q*AfWRKY20* F/R in Table S1), data were collected using the MxPro-Mx3000P system in this study. Each set of experimental data was subjected to three biological replicates, as well as technical replicates.

### 4.6. Subcellular Localization Analysis of AfWRKY20

ORF-specific primers (*AfWRKY20*-F1/R1 in Table S1) were designed using the coding sequence (CDS) of *AfWRKY20* and identified by PCR reaction using the pMD18-T-*AfWRKY20* plasmid as a template. Then, the recovered product of the target band was ligated with the recovered product of the pBI121::*GFP* vector, which had been digested with XbaI and SalI. This resulted in the creation of the two-component plant recombinant expression vector pBI121::*AfWRKY20*::*GFP*, which was placed under the control of the CaMV 35S promoter. In this study, the recombinant vector (35S::*AfWRKY20*::*GFP*) as an experimental group and the empty vector (35S-*GFP*) as a control group were transformed into EHA105 using electroporation. Using the transient transformation method, tobacco plants were successfully transformed by EHA105. The identified strain of EHA105 was injected into the epidermal cells of *N. benthamiana* tobacco. The plants were then incubated in the dark for 12 to 16 h, followed by incubation under natural light for 3 days. Confocal laser fluorescence microscopy (Olympus, Tokyo, Japan) was used to observe the green fluorescence channel, DAPI staining channel, and bright field channel. The superimposed images of the channels were used to confirm the subcellular localization of AfWRKY20 [64]. Finally, it was verified whether it was consistent with the subcellular localization results predicted by Plant-mPLoc.

### 4.7. Analysis of Binding Properties of AfWRKY20 Protein and W-Box Cis-Acting Elements

In this study, EMSA was used to investigate the binding characteristics between *AfWRKY20* and the W-box element. The prokaryotic expression system was used to express and purify the fusion protein GST-AfWRKY20. Glutathione Sepharose 4B (GE) GST affinity chromatography was used to obtain purified GST-AfWRKY20 fusion protein for the subsequent gel blocking assay. Oligonucleotide probes were prepared using synthetic W-box probe primers (W-box F1/R2 in Table S1) and synthesized by Doctor Biology Co. The oligonucleotide probes were labeled with biotin, and a reverse complementary DNA was used as a cold competitive probe (Cold-Pr). The preparation was performed according to the instructions of the EMSA kit (Biyuntian Biological Co., Ltd., Shanghai, China) [65,66].

Four-week-old leaves of *N. benthamiana* tobacco were used for Dual-Luciferase activity assays [67]. The sequences of the W-box (W-box F/R in Table S1) and mW-box (mW-box F/R in Table S1) elements, which consist of three tandem repeats, were synthesized using oligonucleotide sequencing and then seamlessly cloned into the pGreenII 0800-LUC vector. To construct the recombinant plasmid *AfWRKY20*-pGreenII62sk-LUC, specific primers (*AfWRKY20*-62-SK F/R in Table S1) were designed to incorporate the homology arm and SalI and BamHI restriction sites. The recombinant plasmid (Figure S2D) was successfully identified through double enzyme digestion and then electrotransformed into EHA105. W-box-0800, mW-box-0800, pGreenII 0800-LUC empty vectors, pGreenII 62SK, and *AfWRKY20*-62-SK were used to transiently express tobacco leaves. The Agrobacterium hybrid system of pGreenII 0800-LUC vectors and pGreenII 62SK vectors was used as a negative control for transforming *N. benthamiana* tobacco. Leaves were incubated in the dark for 12 h and then exposed to normal light for 3 days. After cutting the leaves, a reaction solution containing 1 mmol/L of the fluorescein substrate D-fluorescein was injected into the wound. The leaves were then left in the dark for 5–7 min. Subsequently, the leaves were examined using a fluorescence imaging system, and the exposure time was adjusted based on the experimental results. Finally, the samples were imaged.

### 4.8. Genetic Transformation and Drought Resistance Analysis of AfWRKY20 Overexpressed Tobacco

#### 4.8.1. Acquisition of *AfWRKY20* Transgenic Tobacco

In this study, tobacco leaves were infected with EHA105 carrying the pBI121::*AfWRKY20*::*GFP* recombinant plasmid. The infected tobacco leaves were then soaked and placed on a tobacco co-medium (1/2MS + As). The leaves were cultured under ambient conditions in the dark for 3 days. Bud differentiation was then induced on tobacco selective differentiation medium containing 50 mg/L of kanamycin. Finally, rooting was induced in a rooting medium (1/2MS + 50 mg/L kanamycin + 250 mg/L carbenicillin), resulting in a T0 generation of *AfWRKY20* transgenic tobacco lines.

In this study, CTAB was used to extract genomic DNA from T0 generation *AfWRKY20* transgenic tobacco lines (#4, #5, #6, and #7) and the WT. *AfWRKY20* overexpressed plants of the T0 generation were identified using PCR molecular biology techniques with specific primers (*AfWRKY20* F1/R1 in Table S1). DNA from the WT was used as the negative control (CK−), the PBI121-*AfWRKY20*-*GFP* plasmid was used as the positive control (CK+), and DNA from the T0 generation *AfWRKY20* transgenic tobacco lines (#4, #5, #6, and #7) was used as the experimental group. The expression of the *AfWRKY20* gene was identified in different transgenic tobacco lines (#4, #5, #6, and #7) using real-time fluorescence quantitative detection technology. RNA was extracted from the transgenic tobacco lines *AfWRKY20* (#4, #5, #6, and #7) as the experimental group, while the WT was used as the control group. The extracted RNA was then reverse transcribed into cDNA. A 100-fold dilution of cDNA was used as the template. The internal reference primer, *AfTubu.*F/R (*AfTubu.*F/R in Table S1), and the specific primer sequence for *AfWRKY20* real-time fluorescence quantification, *AfWRKY20* F/R (q*AfWRKY20* F/R in Table S1), were used. Finally, data were collected using the MxPro-Mx3000P system. Three biological replicates and three technical replicates were performed for each set of experimental data. Finally, the tobacco lines of the experimental group were screened on the 1/2MS medium with kanamycin (50 mg/L) resistance. The aim was to determine if all of the seeds of the transgenic tobacco lines germinated and if the T3 generation *AfWRKY20* transgenic seeds were obtained in a pure state [68].

#### 4.8.2. Tolerance Analysis of *AfWRKY20* Transgenic Tobacco at Germination Stage under Sorbitol and ABA Simulated Drought Stress

The seeds of T3 generation *AfWRKY20* transgenic tobacco (#5, #6, and #7) and the WT tobacco were sterilized and placed in 1/2MS sorbitol solutions at different concentrations (0, 200, and 300 mM) and 1/2MS ABA solutions at different concentrations (0, 2, 2.5, 3, and 5 μM). T3 generation *AfWRKY20* transgenic tobacco seeds (#5, #6, and #7) were used as the experimental groups, while wild-type tobacco seeds (WT) were used as the control group. The experimental and control groups were seeded with 30 seeds on a 1/2MS plate containing different stress-inducing concentrations of sorbitol. The plates were then placed at −4 °C and subjected to vernalization at a low temperature for 3 days. They were then cultured horizontally at 25 °C (8 h light/16 h dark). Germination rate and green leaf rate were measured at the beginning of seed germination in 1/2MS medium under various stress conditions. Three sets of biological replicates and three sets of technical replicates were performed for each experimental set.

Seeds from both the experimental and control groups were placed in 1/2MS medium and cultured vertically until reaching the trifoliate stage. The tobacco plants from both the experimental and control groups, which had the same growth rate, were exposed to various concentrations of sorbitol (0, 200, 250, and 300 mM) and different stress-inducing concentrations of ABA (0, 5, 7.5, and 10 μM) in 1/2MS medium. After 15 days of vertical cultivation at 25 °C (8/16 h-light/dark), we measured the fresh weight and root length under various stress concentrations using ImageJ software (ImageJ Wiki https://imagej.net/ (accessed on 1 Octorber 2022)) for analysis. Three sets of biological replicates and three sets of technical replicates were performed for each experimental group.

#### 4.8.3. Analysis of Photosynthetic Characteristics of *AfWRKY20* Transgenic Tobacco Seedlings under Natural Drought Stress

The T3 generations of *AfWRKY20* transgenic tobacco lines (#5, #6, and #7) mentioned above were vertically cultured until the trifoliate stage. These lines were used as the experimental groups, while the WT tobacco was used as the control group. The tobacco seedlings from both the experimental group and the control group, which had similar growth, were selected for soil cultivation. Sixteen seedlings from each group (4 × 4) were placed in blue pots and cultured at a temperature of 25 °C (8/16 h light/dark cycle) for 1 month. The open chlorophyll fluorescence imaging system was used to investigate the sensitivity of the plant photosystem II response to drought stress in both the experimental and control groups after 0, 10, and 15 days of natural drought treatment. Chlorophyll fluorescence parameters measured by the system included F0 (minimum fluorescence yield in the absence of photosynthetic light), Fm (maximum fluorescence yield in the absence of photosynthetic light), FV/Fm (maximum efficiency of PSII), QL (photochemical quenching coefficient based on the lake model), and NPQ (extent of excess energy dissipation in the form of heat) [69]. At the same time, photographs of tobacco plants were taken under natural light on days 0, 10, and 15 of the natural drought treatment, as well as on the third day after rewatering. In addition, a tobacco chlorophyll fluorescence imager (with a cursor range of 0.2–0.8) was used to capture images in Fv/Fm mode. The survival rate of tobacco was then calculated after 3 days of rehydration [70]. Each experimental group had three biological replicates and three technical replicates.

#### 4.8.4. Determination of Physiological Indices in *AfWRKY20* Transgenic Tobacco under Natural Drought Treatment

*AfWRKY20* transgenic tobacco lines (#5, #6, and #7) and wild-type tobacco lines of the T3 generation, which exhibited consistent growth for 2 months, were selected. These lines were exposed to natural drought conditions for 0, 10, and 15 days, followed by a period of rewatering for 3 days. Fv/Fm values of tobacco were collected using the FluorCam open chlorophyll fluorescence imaging system on day 0 and day 15 of the natural drought treatment and on day 3 of rewatering. The chlorophyll content, MDA content, and SOD activity of tobacco leaves were measured during the natural drought stage and rewatering stage [71]. The tobacco leaves with the most noticeable phenotypic changes were selected for DAB and NBT chemical staining analysis after 15 days of drought treatment [72]. RNA was extracted from transgenic tobacco lines expressing *AfWRKY20* (#5, #6, and #7) after 15 days of drought stress as the experimental group. Wild-type RNA tobacco was used as the control group. The extracted RNA was then reverse transcribed into cDNA.

Using a 100-fold diluted cDNA as a template and *NbActin* F/R (*NbActin* F/R in Table S1) as the internal reference primer, specific primer sequences *NbSOD* F/R, *NbPOD* F/R, and *NbCAT* F/R (*NbSOD* F/R, *NbPOD* F/R, and *NbCAT* F/R in Table S1) were used for real-time fluorescence quantification. Finally, data were collected using the MxPro-Mx3000P system. Three sets of biological replicates and three sets of technical replicates were performed for each experimental set.

### 4.9. Statistical Analysis

All data were processed using the Paired Comparison plot in ORIGIN 2022 software. The following parameters were used in the Paired Comparison plot: Error bar—SD, Mean comparison methods—Tukey, and Significance level—0.05. Lowercase letters (a, b, c, etc.) represent statistical differences with *p* < 0.05.

## Data Availability

Data from this study are available from the corresponding author upon reasonable request.

## Supplementary Materials

The supporting information can be downloaded at: https://www.mdpi.com/article/10.3390/ijms241512231/s1.
