# Supplementary material for: Functional Study of Amorpha fruticosa WRKY20 Gene in Response to Drought Stress
Source: Int J Mol Sci. 2023 Jul 31;24(15):12231. doi: 10.3390/ijms241512231 (PMC10418629; doi:10.3390/ijms241512231)
Supplement: Supplementary file 1 [file ijms-24-12231-s001.zip › ijms-2536184-supplementary.pdf]

Supplementary Material

1 Supplementary Tables

Table S1 Primers needed in the experiment

Primer name	Primer Sequence (5'-3')	Function
AfWRKY20-F1	TCTAGACTATGAACGGAGCTACCACC	Gene Cloning
AfWRKY20-R1	GTCGACATGGACCCGTTAGTATTCT	
AfTubu.F	ACAAGGCGGTTAAGGTTGGT	*Amorpha fruticosa* internal reference genes
AfTubu.R	GTTCTGGGCTTGGTTCCCTT	
q*AfWRKY20* F	GAGTCCGACGTCGTTTTTGG	Real-time quantitative specific primers
q*AfWRKY20* R	TTCAACAGGAGCACTAGCCC	
W-box F1	GGAACTTGACCTTGACCTTAGGGCTGCAGGAATTCG	Probe primers for the W-box of EMSA
W-box R1	CGAATTCCTGCAGCCCCTAAGGTCAAGGTCAAGTCC	
W-box F	GGGCCCCCCCTCGAGGTCGACCGTTGACCGTTGACCGAGTTGACTTTTTTAG	W-box probe primer for the Dual LUC activity
W-box R	CGCTCTAGAACTAGTGGATCCTAAAAAAGTCAACTCGGTCAACGGTCAACGC	

mW-box F	GGGCCCCCCCTCGAGGTCGACCGTAGACGGTAGACGGAGTAGACGTTTTTAG	mW-box probe primer for dual LUC activity
mW-box R	CGCTCTAGAACTAGTGGATCCTAAAAACGTCTACTCCGTCTACCGTCTACGC	
AfWRKY20-62-SK F	CGCTCTAGAACTAGTGGATCCATGAACGGAGCTACCACCAA	Gene primers for Double LUC activity
AfWRKY20-62-SK R	GGTACCGGGCCCCCCCTCGAGGTCGACTCATGGACCCGTTAGTATTCTTC	
NbActin F	CTGCTGGAATTCACGAAACA	Tabacco internal reference genes
NbActin R	GCCACCACCTTGATCTTCAT	
NbSOD F	CTCCTACCGTCGCCAAAT	Real-time quantitative specific primers
NbSOD R	GCCCAACCAAGAGAACCC	
NbCAT F	AGGTACCGCTCATTCACACC	Real-time quantitative specific primers
NbCAT R	AAGCAAGCTTTTGACCCAGA	
NbPOD F	TCCTGGAGTTGTTGCCTTGCTAG	Real-time quantitative specific primers

NbPOD R	GCTTAGTCCTCTTGTCCGTTGCTA

2 Supplementary Figures

A

B

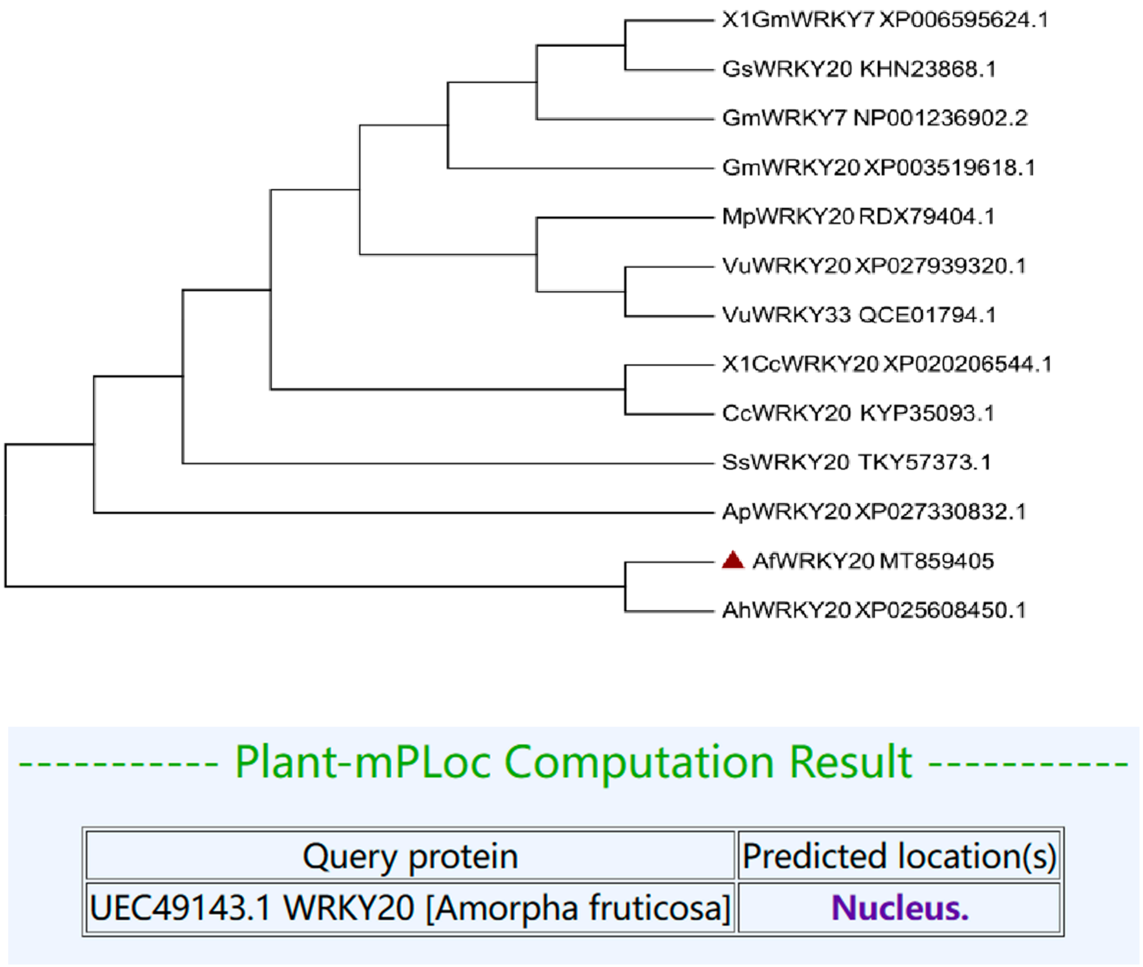

Supplementary Figure S1. (**A**) AfWRKY20 protein phylogenetic tree.Note:Spatholobus s uberectus;Glycine max;Cajanus cajan;Abrus precatorius;Mucuna pruriens;Vigna unguicula ta;Arachis hypogaea;Glycine soja. (**B**) Predicted subcellular localization of AfWRKY20 pr otein.

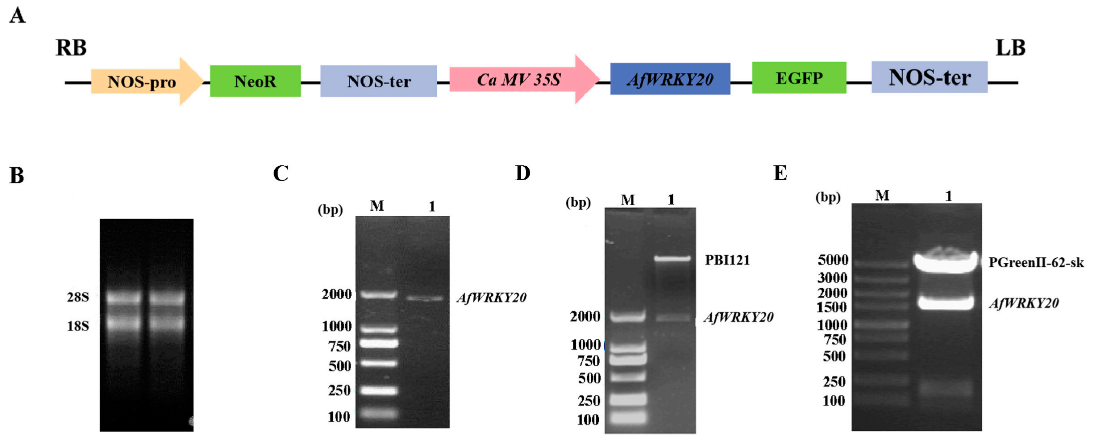

Supplementary Figure S2. (**A**) Diagram of pBI121-*AfWRKY20-GFP*. (**B**) Extraction of total RNA from *Amorpha fruticosa* leaves. (**C**) PCR amplification product electrophoresis detection. (**D**) Identification of pBI121::*AfWRKY20*::*GFP* digest. (**E**) Identification of pGreenII-62-sk::*AfWRKY20* digest.

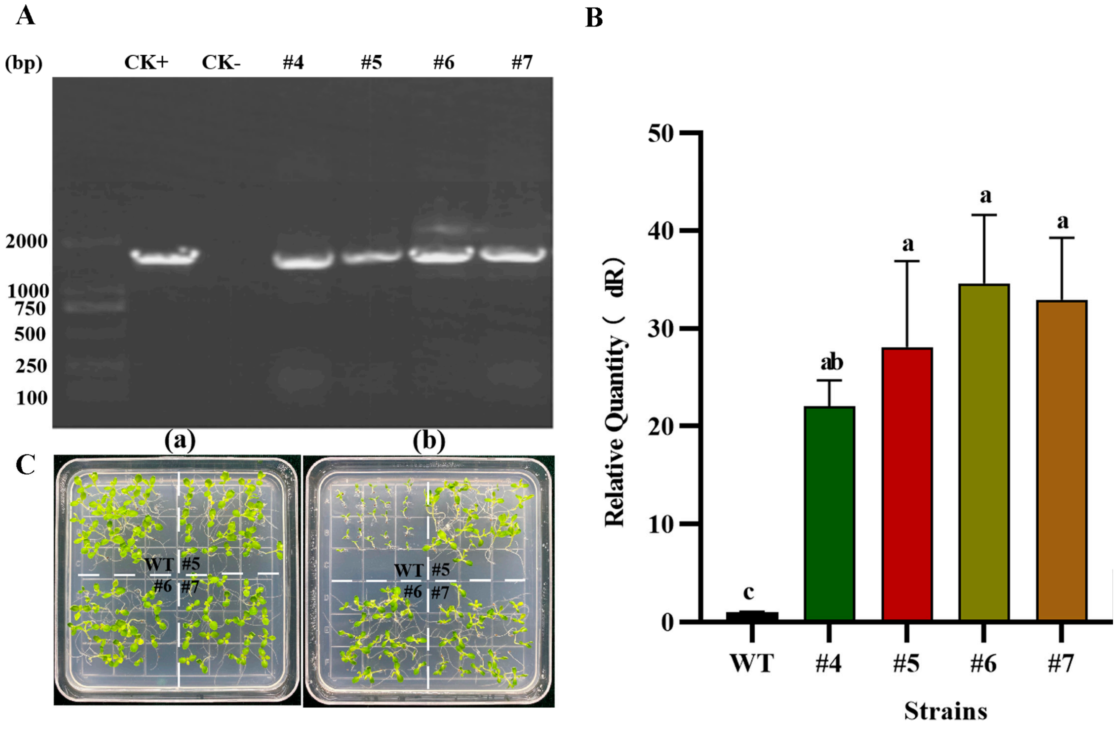

Supplementary Figure S3. (**A**) PCR identification of transgenic plants. (**B**) Relative expression analysis of *AfWRKY20* transgenic tobacco. Lowercase letters (a, b, c, etc.) represent statistical differences with $p < 0.05$. (**C**) (**a**): 1/2 MS medium; (**b**): Kana resistance screening of overexpressed *AfWRKY20* tobacco transformed seedlings.

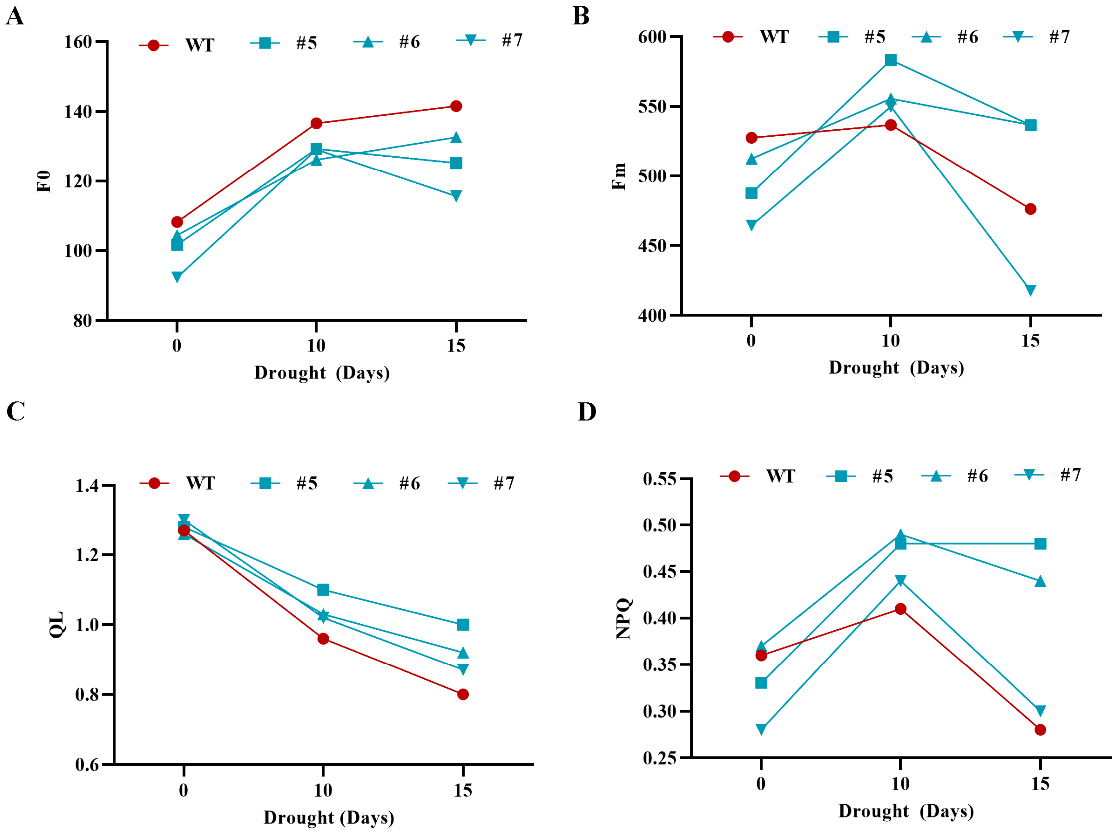

Supplementary Figure S4. Changes in chlorophyll fluorescence parameters of tobacco under natural drought stress for 15 days. (**A**) Variation of the minimum fluorescence yield (F0) in the absence of photosynthetic light. (**B**) Variation of maximum fluorescence yield (Fm) in the absence of photosynthetic light. (**C**) Variation of the proportion of open PSII reaction centers (QL). (**D**) Non-photochemical quenching (NPQ).

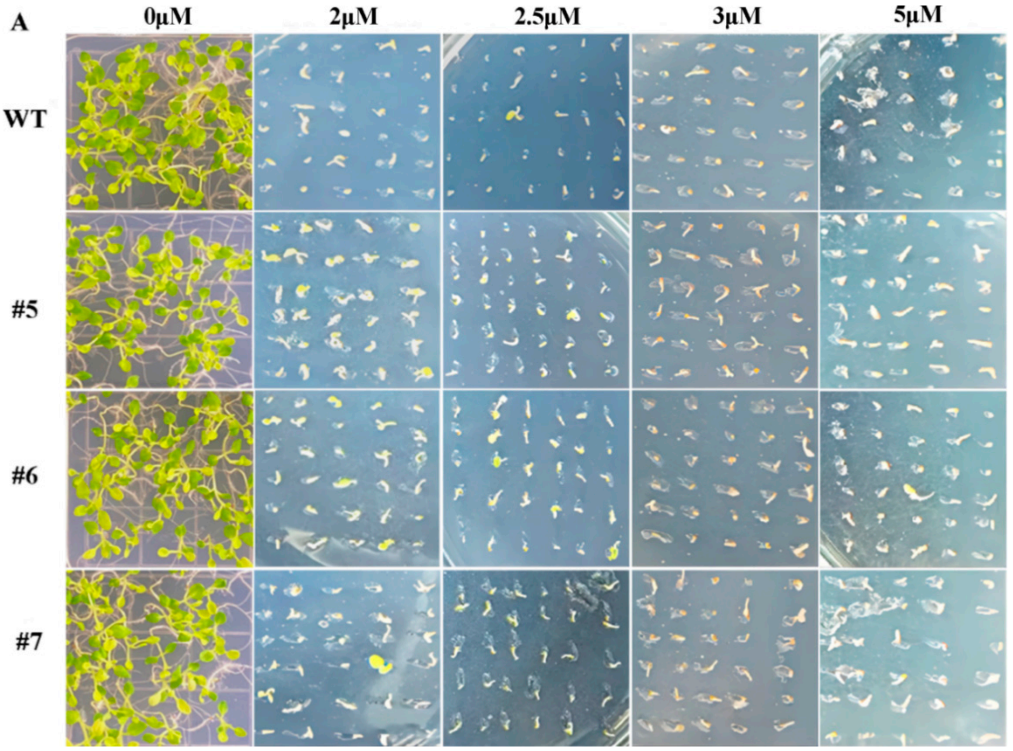

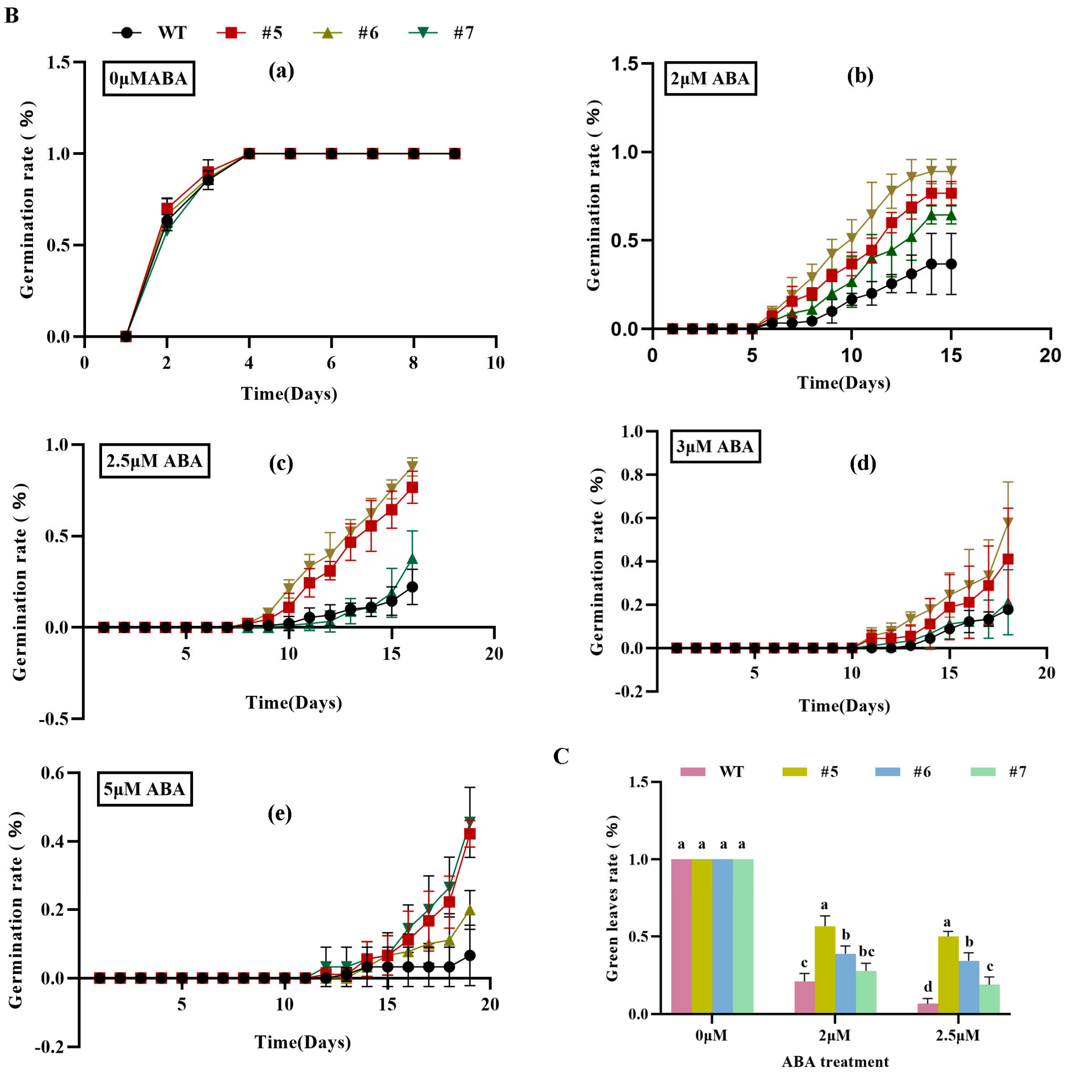

Supplementary Figure S5. Analysis of green leaf rate and germination rate of overexpression strain *AfWRKY20* under different concentrations of ABA stress. (**A**) Germination phenotype of ABA treatment. (**B**) Measurement of plant germination rate. (**a–e**) Trends of tobacco seed germination under different ABA stresses. (**C**) Measurement of plant green leaf rate. Error bars indicate standard errors of three biological replicates, which are significantly different at the $p < 0.05$ level. Lowercase letters (a, b, c, etc.) represent statistical differences with $p < 0.05$.

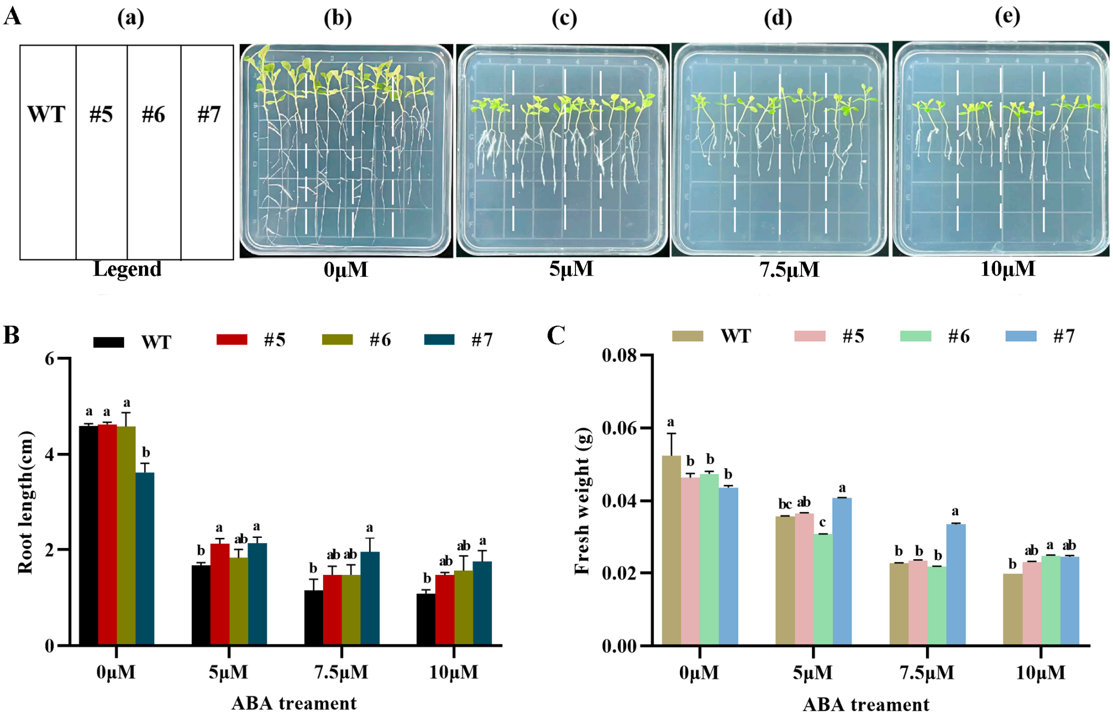

Supplementary Figure S6. Analysis of fresh weight and root length of overexpression strain *AfWRKY20* under different concentrations of ABA stress. WT: wild-type plants; #5, #6 and #7 are three different overexpression strains. (**A**) Phenotype of ABA stress.(a) Schematic diagram of tobacco placement. (**b–e**) Phenotypic map of tobacco after ABA stress at different concentrations. (**B**) Plant root length measurement data. (**C**) Plant fresh weight measurement data. Error lines indicate standard errors of three biological replicates, which are significantly different at the p<0.05 level. Lowercase letters (a, b, c, etc.) represent statistical differences with $p < 0.05$.
